# *Amblyomma tapirellum * (Acari: Ixodidae) collected from tropical forest canopy

**DOI:** 10.12688/f1000research.2-194.v2

**Published:** 2014-01-28

**Authors:** Jose R Loaiza, Matthew J Miller, Eldredge Bermingham, Oris I Sanjur, Patrick A Jansen, Jose R Rovira, Eric Alvarez, Eric Rodriguez, Philip Davis, Larissa C Dutari, James Pecor, Desmond Foley, Meghan Radtke, Montira J Pongsiri

**Affiliations:** 1 Programa Centroamericano de Maestría en Entomología, Universidad de Panamá, Ciudad de Panamá, Panama; 2 Centro de Biodiversidad y Descubrimiento de Drogas, Instituto de Investigaciones Científicas y Servicios de Alta Tecnología, Ciudad de Panamá, Panama; 3 Smithsonian Tropical Research Institute, Panama City, Panama; 4 Department of Biotechnology, Acharya Nagarjuna University, Guntur, India; 5 Walter Reed Biosystematics Unit, Smithsonian Institution, Suitland MD, USA; 6 United States Environmental Protection Agency, Washington DC, USA

## Abstract

Free-ranging ticks are widely known to be restricted to the ground level of vegetation. Here, we document the capture of the tick species *Amblyomma tapirellum* in light traps placed in the forest canopy of Barro Colorado Island, central Panama. A total of forty eight adults and three nymphs were removed from carbon dioxide–octenol baited CDC light traps suspended 20 meters above the ground during surveys for forest canopy mosquitoes. To our knowledge, this represents the first report of questing ticks from the canopy of tropical forests. Our finding suggests a novel ecological relationship between *A. tapirellum *and arboreal mammals, perhaps monkeys that come to the ground to drink or to feed on fallen fruits.

## Introduction

Increasing interest in tick-borne diseases in the Neotropics and particularly in Panama during the last decade has fuelled studies on tick biology, behavior and distribution in this region ^1– 3^. These studies have focused on tick species associated with humans and domesticated animals, likely due to their role as vectors of disease agents ^1, 2, 4^. However, basic knowledge about tick natural history still remains largely unexplored, especially for those taxa that thrive in tropical forests. The tick species *Amblyomma tapirellum* ^5^ predominates over *Amblyomma cajennense* as the primary human tick parasite in lowland forest ecosystems of central Panama and Darien ^6^. Adults of *A. tapirellum* have Baird’s Tapir ( *Tapirus bairdii*) as their primary host, but also opportunistically feed on other wildlife and domesticated mammals ^6, 7^, and also humans ( Table 1). *A. tapirellum* is one of the most common species collected with a cloth dragged through the understory vegetation, but it is not known to be found in arboreal mammals ( Table 1), and in addition, a recent survey of tick occurrence on Panamanian birds found no evidence that this species feeds on birds (Miller *et al., in prep.*). Here, we report *A. tapirellum* collected from mosquito light traps placed in the canopy of old-growth lowland tropical forest on Barro Colorado Island (BCI) in central Panama. To our knowledge, this is the first report of ticks being collected in the canopy of Neotropical forests and highlights the potentially complex ecological relationships of of Neotropical ticks, which as a group, are potential vectors of zonootic diseases in undisrupted forest habitats.

**Table 1.** Reported hosts for *Amblyomma tapirellum* (Dunn, 1933) in Panama.

Order	Family	Species	References
Artiodactyla	Bovidae	*Bos taurus* Linnaeus 1758	Fairchild *et al.* 1966
Chiroptera	Phyllostomidae	*Carollia perspicillata* Linnaeus 1758*	Fairchild *et al.* 1966
Perissodactyla	Equidae	*Equus caballus* Linnaeus 1758	Fairchild 1943
Perissodactyla	Equidae	*Equus caballus*	Fairchild *et al.* 1966
Carnivora	Felidae	*Felis silvestris catus* Linnaeus 1758	Bermúdez *et al.* 2010
Primates	Hominidae	*Homo sapiens* Linnaeus 1758	Fairchild 1943
Primates	Hominidae	*Homo sapiens*	Fairchild *et al.* 1966
Primates	Hominidae	*Homo sapiens*	Bermúdez *et al.* 2012
Pilosa	Myrmecophagidae	*Myrmecophaga tridactyla* Linnaeus 1758	Fairchild *et al.* 1966
Artiodactyla	Cervidae	*Odocoileus virginianus* Zimmermann 1780	Bermúdez *et al.* 2010
Artiodactyla	Tayassuidae	*Pecari tajacu* Linnaeus 1758	Fairchild 1943
Artiodactyla	Tayassuidae	*Pecari tajacu*	Fairchild *et al.* 1966
Perissodactyla	Tapiridae	*Tapirus bairdii* Gill 1865	Fairchild 1943
Perissodactyla	Tapiridae	*Tapirus bairdii*	Fairchild *et al.* 1966
Perissodactyla	Tapiridae	*Tapirus bairdii*	Bermúdez *et al.* 2010

* This record is doubtful as the sample could have been pulled from the body of the collector

## Methods

**Figure 1.**
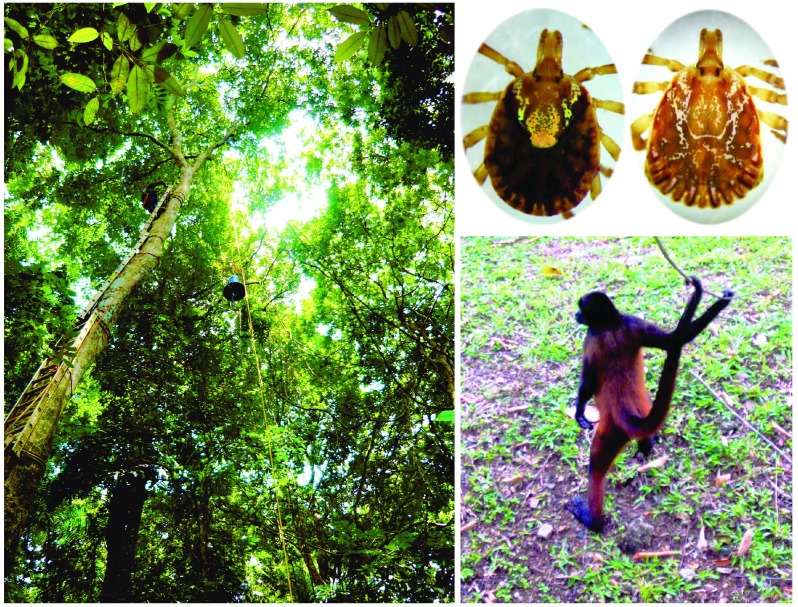
Left side: the set up of one Centers for Disease Control and Prevention miniature light trap in the forest canopy of Barro Colorado Island (BCI). Right upper side: the dorsal view of the scutum of one female (left) and one male (right) of *Amblyomma tapirellum* collected from the forest canopy at BCI. Right lower side: image of an adult of *Ateles geoffroyi panamensis* (Black Spider Monkey) walking freely around the Smithsonian Tropical Research Institute field station at BCI.

Centers for Disease Control and Prevention miniature light traps (CDC-LTs) baited with CO _2_ (dry-ice) and 1-octen-3-ol were placed in areas of old-growth forest on BCI, in the Panama Canal (9.16457 N; -79.86347 W), which has served as a field station for studies of Neotropical flora and fauna for over 100 years. Six traps were placed in the forest canopy (20–30 meters off the ground) and six in the understory (1.5 meters off the ground) for seven consecutive days, every other month, from August 2009 to July 2010 ( Figure 1).

When our field team first discovered the presence of ticks on the outside of CDC-LTs, recognizing that this was a novel occurrence, we carefully reviewed and modified our field protocol to ensure that our observance of host-seeking ticks in the forest canopy was not an artifact of our field methods. To wit, each morning when the traps were lowered, field members, including the senior author, first checked carefully for the presence of ticks on the exterior of each CDC-LT. This was done while the trap was suspended. Any ticks were immediately removed, placed in ethanol and labeled with appropriate metadata (date, trap number, etc.). Subsequently, the netting containing mosquitoes was secured in a plastic box for processing in the indoor laboratory space of BCI. The umbrella and the cylinder containing the fan mechanism of each trap were also taken back to the lab, but the igloo cooler was sealed in a white garbage bag and re-suspended in the mid-canopy (free from by-passers and foliage) during the day. In the evening, CDC-LTs were carried pre-assembled in Rubbermaid-style plastic boxes to the field and were quickly re-assembled in each field site, with loading of the solid CO _2_ as the final step. At no time were either canopy or understory CDC-LTs placed on the ground while they were being serviced in the field. Ticks were counted by trap and preserved as vouchers as part of the ectoparasite - cryological collection of the Smithsonian Tropical Research Institute (STRI).

## Observation

**Figure 2.**
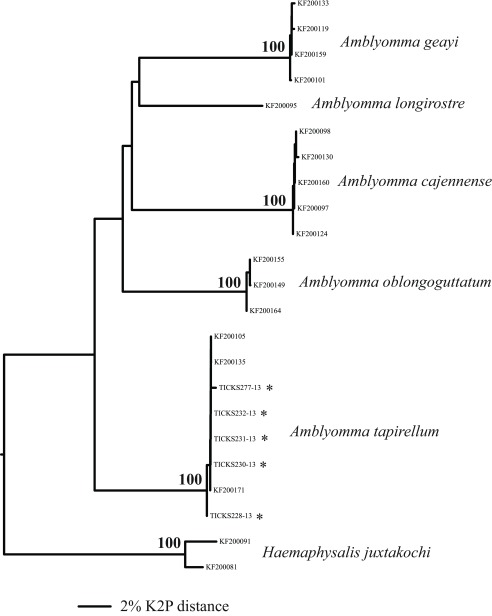
Neighbor-joining tree generated in MEGA 4. Node support (as a percentage) was estimated from 500 bootstrap replicates. Taxa indicated with asterisks (*) represent canopy collected ticks from this study; otherwise tip labels refer to Genbank accession numbers.

Forty eight adults and three nymphs of *A. tapirellum* were collected from CDC-LTs placed in the forest canopy at BCI ( Table 2). All adults were identified using standard taxonomic keys ^6^, while all three nymphs and one adult male and one adult female were confirmed as belonging to *A. tapirellum* based on a neighbor-joining tree generated from reference library of mitochondrial DNA barcoding (COI gene) sequences from Panamanian ticks (Miller *et al., in prep*.). We selected four individuals of *A. geayi*, one of *A. longirostre*, five of *A. cajennense*, three of *A. oblongoguttatum*, three of *A. tapirellum*, and two of *Haemaphysalis juxtakochi* (as an outgroup) to build the tree in MEGA4 ^8^ with group support evaluated via 500 bootstrap replicates ( Figure 2). Mean Kimura 2 parameter (K2P) genetic distance between all five canopy collected ticks and the reference library specimens of *A. tapirellum* was 0.1% (maximum K2P distance 0.6%), well below the typical 2% threshold for interspecific distances for most barcoding studies ^9^. Specimen data, sequences, and sequencing trace files for the five canopy-collected ticks and the 13 reference specimens are archived in the BOLD barcoding database ( dx.doi.org/10.5883/DS-TICKSCAN) and are available on the online global database of DNA barcode sequences ( http://boldsystems.org). Genbank accession numbers for the five canopy ticks generated in this study are: KF370887–KF370891, whereas the Genbank numbers for the adult reference library are: KF200081, KF200091, KF200097, KF200098, KF200101, KF200103, KF200105, KF200119, KF200124, KF200130, KF200133, KF200135, KF200159, KF200160, KF200171.

**Table 2.** Samples of *Amblyomma tapirellum* extracted from CO _2_ – octenol baited Centers for Disease Control and Prevention (CDC) miniature light traps placed in the forest canopy of BCI, central Panama. Each row contains information about the number of specimens collected in one trap during one night. The number of tick positive CDC-LTs out of the total number of canopy traps per month are as following: August (5/42 = 0.119), October (4/42 = 0.095), January (1/42 = 0.023), March (1/42 = 0.023), May (18/42 = 0.418), and July (1/42 = 0.02).

Number of ticks	Life stage and sex	Collection date
2 1 2 1 1	1 male, 1 female Female 1 male, 1 female Male Male	August, 2009 August, 2009 August, 2009 August, 2009 August, 2009
2 1 1 2	1 male, 1 female Female Female Females	October, 2009 October, 2009 October, 2009 October, 2009
1	Female	January, 2010
2	1 male, 1 female	March, 2010
2 2 2 1 2 1 3 3 2 1 2 2 2 3 2 1 1 2	1 male, 1 female 1 male, 1 female Females Male Females Male 1 male, 1 female, 1 nymph 2 males, 1 female 2 nymphs Female 1 male, 1 female 1 male, 1 female 1 male, 1 female 2 males, 1 female 1 male, 1 female Female Male 1 male, 1 female	May, 2010 May, 2010 May, 2010 May, 2010 May, 2010 May, 2010 May, 2010 May, 2010 May, 2010 May, 2010 May, 2010 May, 2010 May, 2010 May, 2010 May, 2010 May, 2010 May, 2010 May, 2010
1	Female	July, 2010

Interestingly, ticks were only extracted from CDC-LTs set up at the canopy level, no ticks were collected from traps at the understory, and only a few canopy traps were positive for ticks ( Table 2). Our findings are unexpected because CDC-LTs are not commonly used to collect ticks, but rather blood-sucking insects such as mosquitoes, sand flies and biting midges. However, they reinforce the notion that ticks use CO _2_ to locate their hosts ^10^. Other *Amblyomma* species have been previously collected with CO _2_ baited traps ^11^, but no study has ever reported host-seeking ticks collected in this fashion from the canopy of a tropical forest. This finding indicates that *A. tapirellum* is not restricted to the ground, but uses both vertical strata (e.g., canopy and ground) to seek hosts. The fact that adults of both sexes as well as nymphs were recovered from canopy traps suggests that *A. tapirellum* can complete its life cycle in the canopy, but this is most likely the result of foresia – the passive movement of one organism by another – by hosts moving vertically. Candidate vectors for movement into the canopy include two monkey species: Mantled Howler Monkey ( *Alouatta palliata*) **and Black Spider Monkey ( *Ateles geoffroyi panamensis*). These two monkey species were often seen near CDC-LTs, and on one occasion, destroyed a trap ( Figure 1). Yet, at present there are no records of *A. tapirellum* collected from these monkeys or any other arboreal mammals in Panama ( Table 1). In addition, the majority of ticks were collected at the beginning of the dry-wet transition period in May 2010 ( Table 2), when ground populations of *A. tapirellum* are quite abundant and monkeys may come to the ground to feed on ripe and over-ripe fruits ^12^. This possibility suggests that an association between arboreal monkeys and ticks is opportunistic, perhaps occurring principally at the peak of the fruiting season ^13^. However, ticks were also collected during August and October of 2009, and so, tick-monkey ground interactions could also be the result of monkey behaviors such as drinking from terrestrial sources or chasing games ^14^. However, we cannot be sure that monkeys are responsible for transporting *A. tapirellum* into the canopy, nor can we explain why ticks were only found on canopy traps and not understory traps; additional studies will be required. Fairchild and collaborators ^6^ noted that *A. geayi* and *A. varium* are practically confined to arboreal sloths. Sloths descend to the ground every three to eight days, dig a hole, defecate, and climb back up into the trees, a behavior that puts the animal at risk if predators are nearby ^15^, and it may also increase the odds of getting ground ticks. Our findings highlight the lack of information on the basic ecology of some species of Neotropical ticks, and argue for an expanded vision of wildlife-tick relationships when planning and conducting disease ecology studies in the Neotropics. Future Neotropical tick surveys in forest areas should include canopy sampling to better understand the bionomics of *A. tapirellum* and its role in pathogen transmission to wildlife.

## References

[1] Bermúdez SE Miranda R: Distribution of ectoparasites of *Canis lupus familiaris* L. (Carnivora: Canidae) from Panama. *Revista MVZ Córdoba.* 2011;16:2274–2282 Reference Source

[2] Bermúdez C SE Castro A Esser H: Ticks (Ixodida) on humans from central Panama, Panama (2010–2011). *Exp Appl Acarol.* 2012;58(1):81–88 10.1007/s10493-012-9564-7 22544074

[3] Murgas IL Castro AM Bermúdez SE: Current status of *Amblyomma ovale* (Acari: Ixodidae) in Panama. *Ticks Tick Borne Dis.* 2013;4(1–2):164–166 10.1016/j.ttbdis.2012.09.002 23128020

[4] Bermúdez SE Eremeeva ME Karpathy SE: Detection and identiﬁcation of rickettsial agents in ticks from domestic mammals in eastern Panama. *J Med Entomol.* 2009;46(4):856–861 10.1603/033.046.0417 19645289

[5] Dunn LH: Two New Species of Ticks from Panama ( *Amblyomma tapirellum* and *A. pecarium*). *Parasitology.* 1933;25(3):353–358 10.1017/S0031182000019557

[6] Fairchild G Kohls G Tipton V: The Ticks of Panama (Acarina: Ixodidae). *In: Wenzel R.L., Tipton V.J. (Eds.), Ectoparasites of Panama* Field Museum of Natural History Chicago, Illinois,1966;pp. 167–219 Reference Source

[7] Fairchild GB: An annotated list of bloodsucking insects, ticks and mites known from Panama. *Am J Trop Med Hygiene.* 1943;23(8):569–591 Reference Source

[8] Kumar S Tamura K Nei M: MEGA3: Integrated software for Molecular Evolutionary Genetics Analysis and sequence alignment. *Brief Bioinform.* 2004;5(2):150–163 10.1093/bib/5.2.150 15260895

[9] Hebert PD Cywinska A Ball SL: Biological identifications through DNA barcodes. *Proc Biol Sci.* 2003;270(1512):313–321 10.1098/rspb.2002.2218 12614582 PMC1691236

[10] Sonenshine DE: Pheromones and other semiochemicals of ticks and their use in tick control. *Parasitology.* 2004;129(suppl):S405–S425 10.1017/S003118200400486X 15938521

[11] Kesinger BJ Allan BF: Efficacy of dry ice-baited traps for sampling *Amblyomma americanum* (Acari: Ixodidae) varies with life stage but not habitat. *J Med Entomol.* 2011;48(3):708–711 10.1603/ME10275 21661336

[12] Leigh EG: Tropical Forest Ecology: A View from Barro Colorado Island. Oxford and New York: Oxford University Press. ISBN 0–19–509602–9/OCLC 3678102.1999 Reference Source

[13] Foster RB: The seasonal rhythm of fruitfall on Barro Colorado Island. In Leigh, E.G., Ran A.S. & Windsor, D. (eds). The ecology of a tropical forest: seasonal rhythms and long-term changes. Smithsonian Institute Press, Washington.1982;151–172 Reference Source

[14] Campbell C Aureli F Chapman C: Terrestrial Behavior of *Ateles* spp. *Int J Primatol.* 2005;26(5):1039–1051 10.1007/s10764-005-6457-1

[15] Voirin JB Kays R Lowman MD: Evidence for Three-Toed Sloth ( *Bradypus variegatus*) predation by spectacled owl ( *Pulsatrix perspicillata*). Edentata no. 8–102009 Reference Source

