# Supplementary material for: Amblyomma tapirellum  (Acari: Ixodidae) collected from tropical forest canopy
Source: F1000Res. 2014 Jan 28;2:194. Originally published 2013 Sep 23. [Version 2] doi: 10.12688/f1000research.2-194.v2 (PMC4095595; doi:10.12688/f1000research.2-194.v2)
Supplement: Peer review history

# Peer review: Referee response for version 2

**Published:** 2014-02-28

**Authors:** Matias Pablo Juan Szabó

**Affiliations:** 1 Institute of Biomedical Sciences and School of Veterinary Medicine, Federal University of Uberlândia, Uberlândia, Brazil

In this new version of the manuscript, authors detailed handling procedures and care taken to avoid accidental trap infestation after the first tick encounter on the mosquito trap. Nonetheless, the phenomenon presented by Jose Loaiza and collaborators is uncommon and deserves further investigation.

However, I suggest removing from the final version the following phrases from the manuscript: “ *This possibility suggests that an association between arboreal monkeys and ticks is opportunistic, perhaps occurring principally at the peak of the fruiting season. However, ticks were also collected during August and October of 2009, and so, tick-monkey ground interactions could also be the result of monkey behaviors such as drinking from terrestrial sources or chasing games.*”  Monkeys as tick carriers up to the canopy is very speculative by itself, explaining how it occurs is excessive speculation. Should this phenomenon occur, many other possibilities should be explored.

I have read this submission. I believe that I have an appropriate level of expertise to confirm that it is of an acceptable scientific standard.

# Peer review: Referee response for version 1

**Published:** 2013-11-08

**Authors:** Matias Pablo Juan Szabó

**Affiliations:** 1 Institute of Biomedical Sciences and School of Veterinary Medicine, Federal University of Uberlândia, Uberlândia, Brazil

The phenomenon presented by Jose Loaiza and collaborators is astonishing by several criterions and thus should be carefully evaluated to dispel any doubt. The mobility described for *Amblyomma tapirellum* is atypical and the explanations rather speculative (which is acceptable considering the absolute lack of knowledge on the matter, as clearly stated by authors). At the same time, finding 48 adult ticks (I will focus on the stage found in the highest numbers) on different occasions in different light traps suspended 20m above the ground indicates a standard.  

One has to consider that for regular access to traps, adult ticks either climbed trees by themselves, fed on an arboreal host and moulted to adults on trees, were taken to the trees as adults by hosts (monkeys) and detached from them or had access to the traps during handling. Apart from the last possibility all others are worthwhile considering and deserve further research. In fact, movement from tree to suspended mosquito trap is, by itself, a challenge for ticks. I am not acquainted with such traps but it seems from the picture that it is held by a string or something similar and thus ticks would have to crawl on a variable length of string to reach the trap. Regardless, before further speculation, it is essential to dismiss trap infestation during handling through review, and careful description of field procedures. Considering the uncommon finding and its ecological importance such information is of paramount importance for the article.

According to the authors, this tick species is quite prevalent on the ground or on ground vegetation. For this reason, I strongly recommend describing the handling of traps in detail. Were they mounted /examined/maintained on the ground at any moment? By personal experience, it is common to find ticks crawling on boxes used to carry dry ice (CO _2_) for tick traps. After finding the first specimens in the traps was any specific care taken to avoid accidental access of ticks to the traps? Were there any differences in the handling of traps in the canopy in comparison to those closer to the ground?

Should these doubts be eliminated in the text, the manuscript will be acceptable for further discussion and publication.

I have read this submission. I believe that I have an appropriate level of expertise to confirm that it is of an acceptable scientific standard, however I have significant reservations, as outlined above.

# Response

**Published:** 2014-01-20

**Authors:** Jose Loaiza

**Affiliations:** Instituto de Investigaciones Cientificas y Servicios de Alta Tecnologia, Panama

- *"According to the authors, this tick species is quite prevalent on the ground or on ground vegetation. For this reason, I strongly recommend describing the handling of traps in detail. Were they mounted/examined/maintained on the ground at any moment? By personal experience, it is common to find ticks crawling on boxes used to carry dry ice (CO2) for tick traps. After finding the first specimens in the traps was any specific care taken to avoid accidental access of ticks to the traps? Were there any differences in the handling of traps in the canopy in comparison to those closer to the ground?"*
  We appreciate Dr. Szabó’s concerns, and as we mentioned above, we apologize for not providing greater detail in our description of our field methods. We hope that the added text will sufficiently describe our field techniques. As we point out, independent confirmation of our surprising finding is on-going in Panama.

We have taken advantage during this revision to fix spelling and grammatical errors in the text and figures:

- In Figure 2, Amblyomma gaeyi was replaced by Amblyomma geayi

# Peer review: Referee response for version 1

**Published:** 2013-11-05

**Authors:** Michael Levin

**Affiliations:** 1 Division of Vector-Borne Diseases, Centers for Disease Control and Prevention, Atlanta, GA, USA

The authors speculate that questing *A. tapirellum* ticks, which are normally feeding primarily on tapirs, could be transported and, moreover, dropped into carbon dioxide baited mosquito traps by primates. (Yes, “Questing” is the appropriate term, which describes a stage in the tick life cycle when it is ready to take a blood-meal, whether it is searching for food actively or passively.) This “delivery of questing ticks” is a tenuous conjecture at best.

Unlike mosquitoes, ixodid ticks feed only once per life stage; they rarely attach to and get transported by unsuitable hosts, on which ticks would not feed. Ticks, that “by mistake” get onto a wrong target – be it a flagging device or an inappropriate host, get off of it within minutes unless they are frozen in dry ice. Ticks which do find a suitable host and begin feeding do not just suddenly drop off, but continue taking a blood-meal for several days until full engorgement (or until the host’s death). *A. tapirellum* has a 3-host life cycle; it means that at each stage engorged (blood-fed) ticks fall off the host into the substrate in order to molt into the next life stage or to lay eggs (unlike in mosquitoes, female ixodid ticks die after just one oviposition). It takes several weeks for an engorged tick to molt and start questing again. Therefore, it is highly implausible that unfed (questing) ticks would be delivered en masse into the traps by any hosts. Engorged ticks, on the other hand, might have been delivered in this way but only if an infested animal crawled into a trap for a rest and fed ticks had time to fall off of it. However, neither spider nor howler monkeys could possibly fit into miniature light traps, not to mention that neither species is known to serve as a host for *A. tapirellum*.

It is much more likely that the appearance of questing *Amblyomma *ticks in mosquito traps resulted from a procedural (trap-handling) artifact. Authors placed their carbon dioxide baited traps either 1.5 meters above the ground (not really “at the ground level”), or 20-30 meters up in the trees. Obviously, traps hanging 1.5 meters above the ground could be serviced right where they were, without contact with the tick-infested ground cover. However, those traps placed 20 meters high would have had to be lowered to the ground daily for checking and replacement of the dry ice, and that would give ticks a chance to crawl onto the traps out of their own volition. Ticks are attracted to carbon dioxide and fast crawling ticks like *Amblyomma* spp. can be collected in large numbers quite quickly. Unfed questing ticks that crawled onto and eventually into dry ice container(s) would be discovered the next day to cause a “sensational discovery”. Uneven distribution and seasonal dynamics of ticks explain why some traps had ticks and others did not. Differences in placement of traps explain why only high-hanging traps caught ticks.

The suppositions advanced in this account can only stem from ignorance regarding ticks, their biology, behavior, and even the standard terminology. It is usually advisable to consider simple explanations of unexpected results prior to making "astounding" claims.

I have read this submission. I believe that I have an appropriate level of expertise to state that I do not consider it to be of an acceptable scientific standard, for reasons outlined above.

# Response

**Published:** 2014-01-20

**Authors:** Jose Loaiza

**Affiliations:** Instituto de Investigaciones Cientificas y Servicios de Alta Tecnologia, Panama

- *"Authors placed their carbon dioxide baited traps either 1.5 meters above the ground (not really “at the ground level”)."*
  To avoid confusion we have changed the text to read “understory.”
   
- *"Obviously, traps hanging 1.5 meters above the ground could be serviced right where they were, without contact with the tick-infested ground cover. However, those traps placed 20 meters high would have had to be lowered to the ground daily for checking and replacement of the dry ice, and that would give ticks a chance to crawl onto the traps out of their own volition. Ticks are attracted to carbon dioxide and fast crawling ticks like Amblyomma spp. can be collected in large numbers quite quickly. Unfed questing ticks that crawled onto and eventually into dry ice container(s) would be discovered the next day to cause a “sensational discovery”. Uneven distribution and seasonal dynamics of ticks explain why some traps had ticks and others did not. Differences in placement of traps explain why only high-hanging traps caught ticks."*
  We appreciate the reviewer calling attention to our lack of detail about our field sampling practice. It is clear that the original draft of our manuscript was without sufficient detail to demonstrate that our finding is not likely to be the consequence of trap-handling artifacts. In fact, when we first found ticks on our traps, our team recognized the novelty of our finding and we took critical steps to ensure that the ticks originated in the canopy and were not accidentally sampled from the ground. We have added the following text to our manuscript:
  "When our field team first discovered the presence of ticks on the outside of CDC-LTs, recognizing that this was a novel occurrence, we carefully reviewed and modified our field protocol to ensure that our observance of host-seeking ticks in the forest canopy was not an artifact of our field methods. To wit, each morning when the traps were lowered, field members, including the senior author, first checked carefully for the presence of ticks on the exterior of each CDC-LT. This was done while the trap was suspended. Any ticks were immediately removed and placed in ethanol and labeled with appropriate metadata (date, trap number, etc.). Subsequently, the netting containing mosquitoes was secured in a plastic box for processing in the indoor laboratory space of Barro Colorado Island. The umbrella and the cylinder containing the fan mechanism of each trap were also taken back to the lab, but the igloo cooler was sealed in a white garbage bag and re-suspended in the mid-canopy (free from by-passers and foliage) during the day. In the evening, CDC-LTs were carried pre-assembled in Rubbermaid-style plastic boxes to the field and were quickly re-assembled in each field site, with loading of the solid CO2 as the final step. At no time were either canopy or understory CDC-LTs placed on the ground while they were being serviced in the field."
   
- *"The suppositions advanced in this account can only stem from ignorance regarding ticks, their biology, behavior, and even the standard terminology. It is usually advisable to consider simple explanations of unexpected results prior to making “astounding” claims."*
  We would like to emphasize that the entire point of F1000Research observational articles is that they are precisely designed to document unexpected results that may improve our understanding of the natural world:
  “Science’s most important breakthroughs, from the discovery of microorganisms to the theory of evolution, have come about through observation. As part of the scientific method, observations are made to record a fact or an occurrence to help either prove or disprove a hypothesis. However, we all know the unpredictable nature of science and the curve balls it can throw” (See more at: http://blog.f1000research.com/2013/09/12/the-observation-article-recording-phenomena-in-scientific-research/#sthash.QP2xbUJj.dpuf )
  Our observations are robust and repeatable. As a research team working at one of the world’s oldest tropical research institutions, we are acutely aware of just how much remains to be discovered in our tropical forests, especially in the extremely inaccessible tropical forest canopy. Given that nymphs and larvae of *A. tapirellum* typically cannot be identified to species in the field (e.g. Bermúdez et al. 2010), and that a Google Scholar search retrieves only 27 articles (including ours) when searching for “ *Amblyomma tapirellum*”, certainly Dr. Levin is not suggesting that our scientific knowledge of the life history of this and other Neotropical ticks species is complete. Fortunately, third party teams are currently actively working in Panama to more fully document the frequency that host-seeking ticks exploit the tropical canopy and confirm our finding. We are grateful for the opportunity given to us by F1000Research to publish our observation that has encouraged those efforts.
   

# Peer review: Referee response for version 1

**Published:** 2013-10-17

**Authors:** Brian Allan, Erin Welsh

**Affiliations:** 1 Department of Entomology, University of Illinois, Urbana, IL, USA; 2 University of Illinois Urbana-Champaign, Champaign, IL, USA

In addition to being notable for describing a unique approach for sampling ticks in Neotropical forests, this article presents the first reported instance of the *Amblyomma tapirellum* tick found in the forest canopy. The authors suggest that this may indicate host transportation of these normally ground-dwelling ticks vertically through the canopy by an unreported host. Though the authors mention that the vertical movement of sloths may lead to ticks being acquired while on the ground, they primarily focus on two candidate host species for the vertical transport of ticks: the Mantled Howler Monkey and the Black Spider Monkey. They hypothesize that the monkeys acquire the ticks on the ground during the peak of fruiting season, and then transport the ticks vertically as they return to the canopy. This is a thoughtful insight that prompts additional questions regarding the natural history of this tick and differences in host acquisition between ground-dwelling and canopy-dwelling populations. One suggestion is to highlight the interesting contradiction that arose from finding no ticks in the ground CDC light traps (CDC-LTs) despite the statement that “ground populations of *A. tapirellum* are quite abundant” during the sampling period. The lack of ticks in ground traps compared to canopy traps could suggest differences in host acquisition strategies between the two environments. In addition, the capture of these ticks in the canopy may indicate that an arboreal life strategy in ticks is a more common phenomenon than previously thought. However, the authors take a different approach regarding stating the article’s significance. Instead of highlighting potentially interesting differences in host acquisition in ticks, the authors focus on implications for disease ecology. Though in this paper these ticks are suggested to be vectors for zoonotic disease, no mention is made of pathogens known to be associated with *A. tapirellum*, which leaves the implications for disease ecology or public health vague and ill-defined. Despite this, the findings in this paper are ecologically interesting and could be framed in a behavioral ecology context to emphasize the importance of finding ground-dwelling ticks actively seeking for hosts in the canopy.

 

Additional, minor revisions could be incorporated to improve the clarity of the paper’s findings. In several instances, *A. tapirellum* is referred to as a vector yet the authors offer no evidence that these ticks transmit a known illness. By definition, a vector is an agent that transmits disease. Additionally, ticks captured by the CDC-LTs are described here as “questing”, but the correct term to describe this behavior is “hunting” or “host seeking”, since “questing” refers to a specific, relatively passive, host acquisition strategy. The authors state that the presence of ticks in the canopy traps “reinforce[s] the notion that ticks use CO­ _2_ to locate their hosts”, a characteristic which is true of many species of ticks. However, this raises the question of why ground traps did not capture ticks, a finding for which no explanations were proposed.

We have read this submission. We believe that we have an appropriate level of expertise to confirm that it is of an acceptable scientific standard.

# Response

**Published:** 2014-01-20

**Authors:** Jose Loaiza

**Affiliations:** Instituto de Investigaciones Cientificas y Servicios de Alta Tecnologia, Panama

- *"In several instances, A. tapirellum is referred to as a vector yet the authors offer no evidence that these ticks transmit a known illness."*
  The sentence in the Introduction now reads: “…highlights the potentially complex ecological relationships of Neotropical ticks, which as a group, are potential vectors of zoonotic disease in undisrupted forest habitats.”
   
- *"Additionally, ticks captured by the CDC-LTs are described here as “questing”, but the correct term to describe this behavior is “hunting” or “host seeking”, since “questing” refers to a specific, relatively passive, host acquisition strategy"*
  References to “questing” were changed to “host-seeking”.
   
- *"The authors state that the presence of ticks in the canopy traps “reinforce[s] the notion that ticks use CO­2 to locate their hosts”, a characteristic which is true of many species of ticks. However, this raises the question of why ground traps did not capture ticks, a finding for which no explanations were proposed"*
  We agree that it is curious that we did not find ticks on the understory traps. However, as we don’t have a plausible explanation, we defer from additional speculation, as it is apparent that simply reporting our finding has created enough controversy without additional conjecture.
